# Hsp70 - a biomarker for tumor detection and monitoring of outcome of radiation therapy in patients with squamous cell carcinoma of the head and neck

**DOI:** 10.1186/1748-717X-9-131

**Published:** 2014-06-09

**Authors:** Mathias Gehrmann, Hanno M Specht, Christine Bayer, Markus Brandstetter, Barbara Chizzali, Marciana Duma, Stephanie Breuninger, Kathrin Hube, Sophie Lehnerer, Valerie van Phi, Eva Sage, Thomas E Schmid, Michael Sedelmayr, Daniela Schilling, Wolfgang Sievert, Stefan Stangl, Gabriele Multhoff

**Affiliations:** 1 Department of Radiation Oncology, Klinikum rechts der Isar, Technische Universität München, Munich, Germany; 2 Hals-Nasen-Ohrenklinik und Poliklinik, Klinikum rechts der Isar, Technische Universität München, Munich, Germany; 3 Clinical Cooperation Group - “Innate Immunity in Tumor Biology”, Helmholtz Zentrum münchen, Munich, Germany

**Keywords:** Hsp70, Tumor, Biomarker, Radiation therapy

## Abstract

**Background:**

Tumor but not normal cells frequently overexpress heat shock protein 70 (Hsp70) and present it on their cell surface (mHsp70) from where it can be actively released. Therefore, membrane (mHsp70) and soluble Hsp70 (sHsp70) were investigated as potential tumor biomarkers and for monitoring the outcome of radiation therapy.

**Methods:**

Biopsies and blood were collected from patients with squamous cell carcinoma of the head and neck (SCCHN) at different time points (before, during therapy and in the follow-up period). Hsp70 membrane expression was determined on single cell suspensions of tumor biopsies and reference tissues by flow cytometry, sHsp70 protein and antibody levels were determined in the serum of patients and healthy donors by ELISA and NK cell markers that are related to the presence of sHsp70 were analyzed in the patient’s peripheral blood lymphocytes (PBL).

**Results:**

Tumor biopsies exhibited significantly increased mHsp70 expression levels compared to the reference tissue. Soluble Hsp70 levels were significantly higher in SCCHN patients compared to healthy human volunteers and high mHsp70 expression levels on tumor cells were associated with high sHsp70 levels in the serum of patients. Following surgery and radiotherapy sHsp70 levels in patients dropped in patients without tumor relapse in the follow-up period. In contrast to sHsp70 protein, anti-Hsp70 antibody levels remained nearly unaltered in the serum of SCCHN patients before and after therapy. Furthermore, sHsp70 protein but not anti-Hsp70 antibody levels were found to be associated with the tumor volume in SCCHN patients before start of therapy. The expression densities of the activatory NK cell markers CD56, CD94, NKG2D, NKp30, Nkp44, and NKp46 differed in patients following therapeutic intervention. A significant increase in the density of NKG2D was observed in SCCHN patients in the follow-up period after surgery and radiotherapy.

**Conclusion:**

We suggest sHsp70 as a potential biomarker for detecting tumors and for monitoring the clinical outcome of radiotherapy in SCCHN patients.

## Introduction

Biomarkers are getting more and more important for a better prognosis and for and improved follow-up of a therapy as recently shown for gastro-intestinal tumors [1,2]. Optimal biomarkers should be presented on the cell surface of a broad variety of tumor cells but not on normal cells and should be released in a tumor-specific manner. In order to use biomarkers for the prediction of outcome it is important that the biomarkers remain stable during therapeutic intervention. The presence of biomarkers in the urine or blood of a patient allows repeated tests with a minimal invasive intervention.

For a long time heat shock proteins including Hsp70 and auto-antibodies directed against them have been shown to provide useful biomarkers for the prediction of prostate cancer [3] and squamous cell carcinoma of the oesophagus [4]. Elevated heat shock protein levels have been detected in many different types of cancer [5]. Nevertheless, the approaches have not been validated sufficiently and thus are yet not in clinical use. Hsp70 the major stress-inducible member of the HSP70 family has the potential for being a predictive marker molecule for several reasons. Hsp70 is overexpressed in many different tumor types; it is protecting cells from DNA damage [6], and exerts anti-apoptotic functions [7]. Overexpressed inside cells, Hsp70 is transported to the cell membrane and also exported into the extracellular space [8]. The transport routes are not following the classical transport routes through the ER and Golgi apparatus but instead are mediated through endosomal and lysosomal vesicles [9]. Only a small proportion of Hsp70 is released as free Hsp70 by necrotic cell death. Our findings demonstrate that the majority of extracellular Hsp70 is bound to small lipid vesicles [9]. Theses vesicles are actively released by tumor cells presumably for signaling purposes. It already has been shown that released Hsp70 can stimulate the adaptive immune system [10]. NK cells also recognize Hsp70, bind it and thus can be activated [11]. Following activation, NK cells actively migrate towards Hsp70 positive tumor cells, and kill them via the release of the cytotoxic enzyme granzyme B [12].

Extracellular Hsp70 can be detected by ELISA. Recent results of our group show that Hsp70 can be detected in the plasma of mice bearing human xenograft tumors [13]. Additionally, the plasma Hsp70 levels correlate with the tumor burden of mice assuming the potential of Hsp70 as predictive tumor marker.

Since Hsp70 can be released by human tumor cells with a membrane Hsp70-positive phenotype, we studied the diagnostic impact of sHsp70 in serum of patients suffering from SCCHN. Serum levels of sHsp70 were assessed in patients before and at different time points after radiation therapy and compared to that of healthy controls. In order to study the role of sHsp70 as a potential stimulator of NK cells we concomitantly studied the expression of activatory NK cell markers on the PBL of the patients.

## Patients and methods

### Patients

In total, 21 (20 male, 1 female) patients suffering from tumors localized in the oral cavity (5 cases), larynx (6), oro/hypopharynx (2), oropharynx (7), sinus (1) and 28 age-matched healthy human volunteers were enrolled into the study (Tables 1 and 2). The mean age of all patients was 66.7 years (range 38–78) with a median of 66.0 years and that of healthy individuals was 61.0 (mean 47–74) with a mean of 61.5 years, respectively (Table 1). The clinicopathological characteristics and the treatment regime of each individual patient are summarized in Table 2. Tumor volume was determined by manually contouring the gross tumor volume (GTV) based on contrast enhanced computer tomography scans using iPlan planning software (Brainlab, Feldkirchen, Germany). Total radiation doses following tumor excision were 64 Gy (11 patients), and 60 Gy (2 patients), respectively. One patient received a definitive radiation with a total dose of 70 Gy, and 7 patients received no adjuvant radiation therapy following complete tumor resection. All patients and healthy human individuals provided written informed consent for the collection of blood and tumor biopsies which was approved by the Ethics Committee of the Klinikum rechts der Isar der Technischen Universität München. Blood was collected from untreated tumor patients before surgery and adjuvant radiotherapy and at indicated time points after tumor excision and radiotherapy by venepuncture. 9 ml EDTA-blood and 9 ml serum were collected in S-Monovettes (Sarstedt, Nümbrecht, Germany).

**Table 1:** Age and gender of SCCHN patients and healthy human volunteers

		**Healthy controls**	**SCCHN patients**
Number (n)		28	21
Gender (M/F)		12/16	20/1
Age	Mean (Range)	61.0 (47–74)	66.7 (38–78)
	SD	7.0	9.2
	Median	61.5	66.0

*Abbreviations*: *M* male, *F* female, *SD* standard deviation, *SCCHN* Squamous Cell Carcinoma of the Head and Neck.

**Table 2:** Clinicopathological characteristics of all SCCHN patients

**Patient #**	**Tumor location**	**Stadium**			**Grading**	**Therapy**	
		**T**	**N**	**M**		**Surgery**	**RTx dose (Gy)**
1	Oral cavity	2	2 cd	0	2	+	0
2	Larynx	4	2b	0	3	+	64
3	Oral Cavity	1	0	0	2	+	60
4	Oro/Hypopharynx	4	2a	0	2	+	64
5	Oropharynx	3	2	0	2	-	70
6	Oropharynx	1	2b	0	3	+	64
7	Oral Cavity	1	2a	0	2	+	64
8	Oro/Hypopharynx	1	0	0	2	+	64
9	Oral Cavity	1	0	0	2	+	64
10	Oral Cavity	1	0	0	3	+	0
11	Oropharynx	2	2a	0	2	+	0
12	Larynx	2	2b	0	3	+	0
13	Oropharynx	4	0	0	2	+	64
14	Oropharynx	2	0	0	3	+	64
15	Larynx	3	0	0	3	+	64
16	Larynx	4	1	0	3	+	0
17	Oropharynx	2	2b	0	3	+	0
18	Oropharynx	2	1	0	2	+	64
19	Larynx	2	1	0	2	+	64
20	Sinus	2	0	0	3	+	60
21	Larynx	4	0	0	3	+	0

### Tumor biopsies

Biopsies in the size range of a few mm^3^ were taken during tumor excision. As a reference, connective tissues derived from 7 tumor-free donors were collected. Single cell suspensions from freshly isolated tumor biopsies were isolated by mechanical disintegration and by forcing the cell suspension through a sterile mesh (75 μm) to obtain single cell suspensions according to a method described previously [14].

### Serum

Blood was centrifuged at 750 × g at room temperature (RT) for 10 min the supernatant containing the serum was removed, mixed, and stored at −80°C in aliquots. Sera were used for experiments after thawing only once. Control serum samples were collected from age-matched healthy human volunteers (Table 1).

### ELISA assays

Total sHsp70 levels in serum samples of humans were measured using a modified Hsp70 immunoassay (Duoset, DYC1663, R&D Systems, Minneapolis, MN, USA). The ELISA is designed to detect human Hsp70 in buffer. All serum samples were analyzed in three independent experiments in duplicates.

Anti-Hsp70 antibodies in the serum were detected using a sandwich ELISA. Briefly, Hsp70 protein (ADI-NSP-555, Enzo Life Sciences, Farmingdale, NY, USA) was coated onto MaxiSorp 96-well plates (NuncNalgene, Thermo Fisher Scientific, Waltham, MA, USA). After incubation with serum the bound antibodies were detected by incubation with HRP-conjugated anti-human Ig followed by HRP-substrate (DY999, R&D Systems, Minneapolis, MN, USA).

### Flow cytometry

Single cells from freshly isolated tumor biopsies were prepared by mechanical disintegration of the tissue, as described previously [14]. 1 × 10^5^ cells were washed once with 10% FCS in PBS and incubated with a FITC-conjugated mouse monoclonal antibody specific for membrane-bound Hsp70 (cmHsp70.1, IgG1, multimmune GmbH, Munich, Germany) or a FITC-labeled isotype-matched IgG1 negative control antibody (345815, BD Biosciences, Franklin Lakes, NJ, USA) on ice in the dark for 30 min. After washing, propidium iodide was added and viable cells were analyzed on a FACSCalibur flow cytometer (BD Biosciences). The percentage of cells stained with an isotype-matched control antibody was subtracted from the percentage of mHsp70 positive cells.

NK cell markers were determined in the peripheral blood of the patients by flow cytometry as described previously [15]. After incubation with fluorescence-conjugated antibodies (CD56, CD94, CD3, CD94, NKG2D, NKp30, NKp44, NKp46), or the appropriate mouse isotype-matched control antibodies, erythrocytes were lysed using the FACS lysing Solution (BD Biosciences) according the manufacturer’s instructions. After washing, lymphocytes were analyzed on a FACSCalibur flow cytometer (BD Biosciences). The NK cells were gated according to their CD56 positivity and CD3 negativity. The percentage of cells stained with an isotype-matched control antibody was subtracted from the percentage of antibody-positively stained cells.

### Statistical analysis

Statistical analysis was performed using SigmaPlot software delivered by Systat Software Inc. (San Jose, CA, USA). Results for the levels of mHsp70, sHsp70, or anti-Hsp70 antibodies are presented as standard box plots with boundaries indicating the 25^th^ and the 75^th^ percentile. The line inside boxes indicates the median and the whiskers indicate the 10^th^ and 90^th^ percentile, respectively. All outliers are shown. The square of the coefficient of correlation parameter R^2^ and linear regression analysis were used to determine the relationships between variables. For comparison between groups of data the student’s *t*-test was used to evaluate differences. *p*-values <0.05 were considered to be statistically significant.

## Results

### Hsp70 membrane expression on tumor biopsies of SCCHN patients

**Figure 1:**
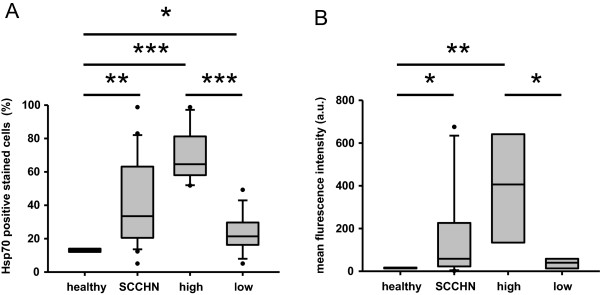
**Hsp70 membrane expression on biopsies of SCCHN patients. A**. The cells stained with FITC-labeled cmHsp70.1 monoclonal antibody corrected with IgG1 isotype-matched control antibody are depicted in box plots. Data from patients’ tumor cells (n = 21) were divided into low (n = 10) and high (n = 11) mHsp70 expressing cells. Single cells from healthy donors (n = 7) derived from connective tissues were used as controls. **B**. Mean fluorescence intensity from the same data set as shown in A. Standard box plots are shown with boundaries indicating the 25^th^ and the 75^th^ percentile. The line inside boxes indicates the median and the whiskers indicate the 10^th^ and 90^th^ percentile, respectively. All outliers are included into the graphs. (*p < 0.05, **p < 0.01, ***p < 0.001).

Tumor biopsies were obtained from patients with SCCHN by tumor resection (n = 21) and reference connective tissues of tumor-free donors (n = 7). The Hsp70 membrane status (mHsp70) was determined on viable single cell suspensions by flow cytometric (FACS) analysis using cmHsp70.1 monoclonal antibody. The mean percentage of Hsp70 positive cells was significantly higher in tumor patients compared to that of the tissue of 7 control tissues which were prepared in parallel (mean 38% vs. 13%) (Figure 1A). The percentage of mHsp70 positive cells in patient biopsies ranged between 5 to 100%. According to their Hsp70 membrane status, the results of the patient samples could be divided into two subgroups. The mean percentage of mHsp70 positive cells in the group with a high Hsp70 expression (n = 11) was around 83% and that of the low Hsp70 expressing group (n = 1) was 20% (Figure 1A, boxplots on the right hand side). The mean fluorescence intensity values correlated with the data of the percentage of Hsp70 membrane positive cells (Figure 1B).

### sHsp70 serum levels in patients with SCCHN

**Figure 2:**
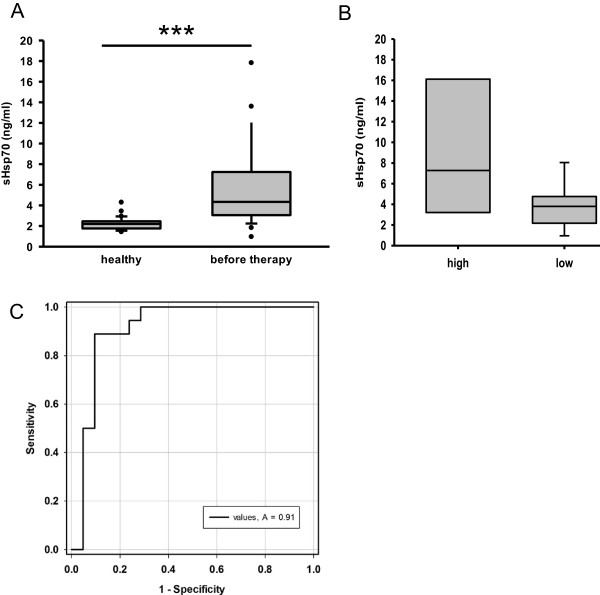
**sHsp70 serum levels in blood of patients with SCCHN. A**. sHsp70 concentrations in sera of patients (n = 21) before therapy compared to healthy donors (n = 28). **B**. sHsp70 serum data from patients were separated into two groups (high n = 11, low n = 10) reflecting the mHsp70 membrane expression as measured in A. **C**. Standard box plots are shown with boundaries indicating the 25^th^ and the 75^th^ percentile. The line inside boxes indicates the median and the whiskers indicate the 10^th^ and 90^th^ percentile, respectively. All outliers are shown. (***p < 0.001) **C**. ROC curve of data from (A) were attained by SigmaPlot software showing an AUC of 0.9101 (*p* < 0.0001, CI 95%). The cut-off level was 2.5 ng/ml with a sensitivity of 89% and a specificity of 91%). Abbr.: ROC, receiver operating characteristic; AUC, area under the curve; CI, confidence interval.

To address the question whether the membrane Hsp70 expression correlates with the soluble Hsp70 (sHsp70) serum levels, sHsp70 levels were determined in serum of SCCHN patients before start of any therapy. The mean sHsp70 levels were significantly elevated in SCCHN patients (5.3 ± 4.1 ng/ml, n = 21) compared to age-matched healthy human volunteers (2.2 ± 0.6 ng/ml, n = 28) (Figure 2A). Patients with tumors that exhibit a high Hsp70 membrane (mHsp70) expression (Figure 1, high) also exhibited higher sHsp70 serum levels (7.2 ± 6.7 ng/ml, n = 11) than patients with a low (Figure 1, low) Hsp70 membrane expression (3.4 ± 1.2 ng/ml, n = 10) (Figure 2B).

Receiver operating characteristic (ROC) curve analysis was performed to explore the potential role of sHsp70 in serum as a diagnostic tumor biomarker for SCCHN. The sHsp70 levels of healthy donors were compared to that of SCCHN patients. The ROC curve reveals an area under the curve (AUC) of 0.91 (p < 0.0001, CI 95%). This means that the cut-off level was 2.5 ng/ml with a sensitivity of 89% and with a specificity of 91% for sHsp70 as a tumor biomarker.

### Time-dependent alterations in sHsp70 and anti-Hsp70 antibody levels in the serum of SCCHN patients

**Figure 3:**
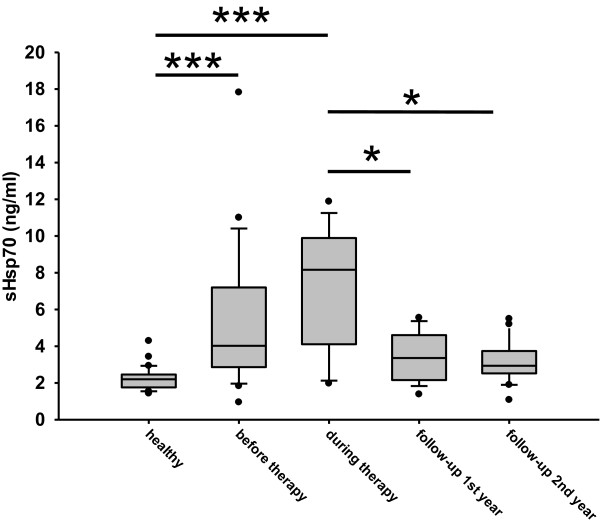
**sHsp70 levels in the serum of SCCHN patients untreated and after therapy.** Blood was collected from patients with SCCHN. sHsp70 concentration was measured with ELISA in three independent experiments in duplicates. Standard box plots are shown with boundaries indicating the 25^th^ and the 75^th^ percentile. The line inside boxes indicates the median and the whiskers indicate the 10^th^ and 90^th^ percentile, respectively. All outliers are shown in the graph. (healthy n = 28, before therapy n = 21, during therapy n = 10, follow-up 1^st^ year n = 9, follow-up 2^nd^ year n = 14). The reason for the different n numbers is due to some missing blood samples during the course of disease. (*p < 0.05, ***p < 0.001).

**Figure 4:**
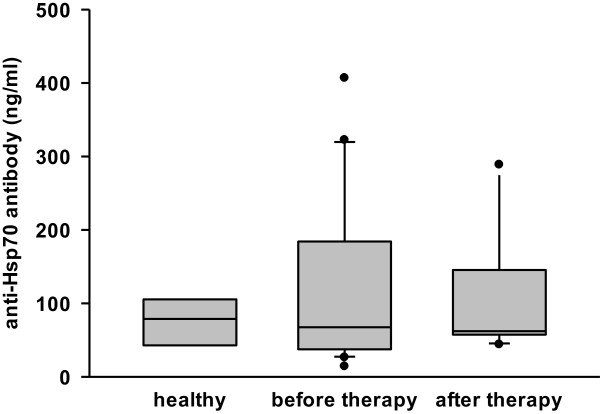
**Anti-Hsp70 antibody levels in the serum of patients before, and at indicated time-points after radiation therapy.**Time course of anti-Hsp70 antibodies in the serum of patients with SCCHN tumors before and after therapy. Blood was collected and anti-Hsp70 antibodies were determined by anti-Hsp70 antibody ELISA. Standard box plots are shown with boundaries indicating the 25^th^ and the 75^th^ percentile. The line inside boxes indicates the median and the whiskers indicate the 10^th^ and 90^th^ percentile, respectively. All outliers are shown in the graph. (healthy n = 4, patients before therapy n = 16, patients after therapy at least 6 weeks after radiation therapy n = 6).

An ideal tumor biomarker should be able to predict clinical responses to therapy, such as radiation therapy or surgery. Soluble Hsp70 levels were found to be significantly elevated in tumor patients before therapy (5.3 ± 4.1 ng/ml, n = 21) compared to healthy donors (2.2 ± 0.6 ng/ml, n = 28). The levels remained elevated up to 6 weeks after tumor resection (7.0 ± 3.6 ng/ml, n = 10) (Figure 3, during therapy). In the first and second follow-up year after radiotherapy sHsp70 levels dropped to 3.4 ± 1.4 ng/ml (n = 9) and 3.1 ± 1.1 ng/ml (n = 14), respectively (Figure 3, follow-up 1st year, follow-up 2nd year).The mean anti-Hsp70 antibody concentrations were not significantly different in tumor patients before therapy (122 ± 119 ng/ml, n = 16) and in healthy human volunteers (84 ± 64 ng/ml), as shown in Figure 4. Also after therapy (surgery/radiotherapy) the mean anti-Hsp70 antibody concentrations in the serum did not change significantly (140 ± 119 ng/ml, n = 6).

### Correlation of sHsp70 protein and anti-Hsp70 antibody levels with the tumor volume of the patients with SCCHN

**Figure 5:**
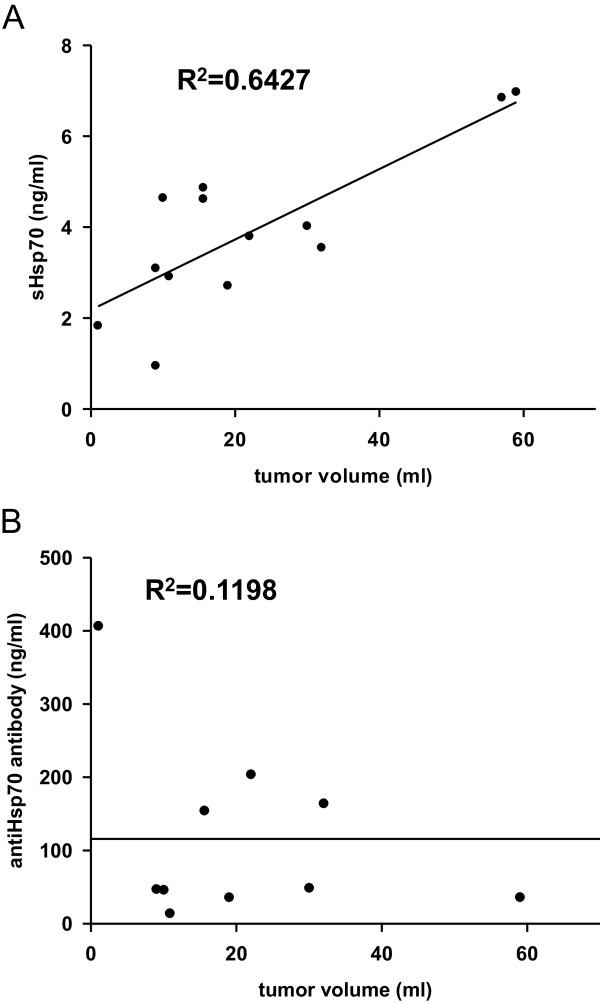
**Correlation of tumor volume with Hsp70 protein and anti-Hsp70 antibody serum levels in patients with SCCHN.** Tumor volumes were determined by manually contouring the gross tumor volume (GTV) of patients based on contrast enhanced computer tomography scans before radiation therapy. The assessed tumor volumes in ml were associated with the sHsp70 serum (**A**, n = 13) and anti-Hsp70 antibody (**B**, n = 10) levels as determined by ELISA. Linear regression was determined by SigmaPlot statistical software.

To evaluate whether the serum levels of sHsp70 protein or anti-Hsp70 antibodies correlate with tumor volumes, the tumor volumes were determined by using CT images. The tumor size in the cohort of SCCHN patients (n = 13) before radiation therapy ranged from below 10 ml up to > 40 ml (the maximum tumor size was 65 ml). Although not statistically significant, the detected serum sHsp70 levels in patients with larger tumor volumes were higher than in patients with smaller tumors (R^2^ = 0.6427) (Figure 5A), whereas the anti-Hsp70 antibody levels in the serum showed no association with the tumor volume (R^2^ = 0.1198) (Figure 5B).

### Expression of activatory NK cell receptors on PBL of SCCHN patients

**Figure 6:**
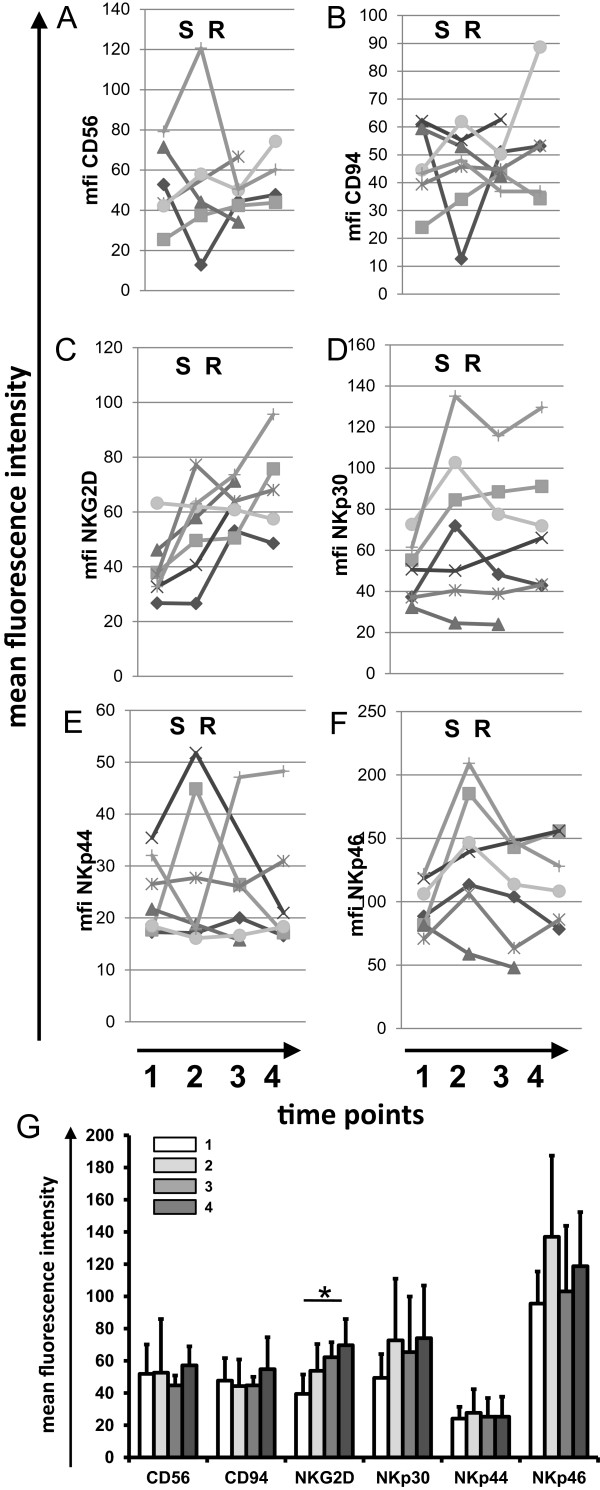
**NK cell activatory markers on peripheral blood lymphocytes (PBL) of SCCHN patients before and at indicated time-points after therapy.** EDTA blood samples from selected patients (n = 7) were collected at indicated time points before therapy (1), after tumor resection before start of radiation therapy (2), in the 1^st^ year of follow-up (3), and in the 2^nd^ year of follow-up (4). The time points of surgery and radiation are indicated with S and R in the upper part of each graph. NK cells were stained by using the lysis-wash method and analyzed by flow cytometry. **A-F**. CD56-positive and CD3-negative NK cells were gated and analyzed. Mean fluorescence intensity (mfi) of CD56, CD94, NKG2D, NKp30, NKp44, NKp46 were determined. Time-curves are shown for individual patients. **G**. Mean fluorescence intensity values of all markers of all patients with error bars and standard deviations (n = 7). Samples were taken before therapy (1), after tumor resection before start of radiation therapy (2), in the 1st year of follow-up (3), and in the 2nd year of follow-up (4) * p<0.05.

In order to test the relevance of the sHsp70 with respect to the stimulation of an NK cell mediated immune response, the expression of activatory NK cell markers was assessed in the blood samples of selected patients before tumor excision and at indicated later time points after radiotherapy. The expression density of the markers CD56, CD94, NKp30, NKp44, and NKp46 varied in the tested patients before, during therapy and within the follow-up period (Figure 6A-F). With respect to the receptor NKG2D, the expression density gradually increased throughout the whole monitoring period in all patients (Figure 6C). A significant increase in the expression density of NKG2D was observed in the blood of patients before therapy (1) and after the second follow-up year (4) (Figure 6G, n = 7).

## Discussion

Tumor biomarkers are useful tools for tumor detection and for monitoring the clinical outcome. Instead of tumor biopsies, which are difficult to obtain, blood samples that can be taken regularly by minimal invasive methods qualify much better to measure clinical outcome. Heat shock proteins are frequently overexpressed in tumor cells and thus are reported as biomarkers [5], in prostate [3], and in pancreatic carcinomas [16]. It also has been shown previously that elevated Hsp70 levels can act as a read-out for the efficacy of Hsp90 inhibitor-based therapies [17]. Also autoantibodies directed against Hsp70 in squamous cell carcinoma [4] are discussed as potential biomarkers.

As already demonstrated for other tumor entities [14] also SCCHN tumors are frequently found to be Hsp70 membrane-positive (Figure 1). Furthermore, in our relatively small cohort of 21 patients two subgroups could be identified that differ in their Hsp70 membrane positivity. Eleven patients showed a high and ten patients a low Hsp70 membrane expression. A longer follow-up of the patients and the testing of a larger cohort of patients will elucidate whether differences in the membrane Hsp70 density has an impact on diagnosis or clinical outcome. Furthermore, we could show that the Hsp70 membrane expression on the tumor biopsy was associated with increased sHsp70 serum levels (Figure 2). Elevated sHsp70 levels were detectable up to six weeks after tumor resection (Figure 3). A slight increase in sHsp70 serum levels might be explained by sHsp70 which is released by dying cells during therapy. Within the first and second year follow-up period the sHsp70 levels dropped significantly in patients who did not relapse. In contrast to the sHsp70 protein the levels of anti-Hsp70 antibodies were only slightly increased in tumor patients compared to that of healthy individuals.

We could show that in a well-established FaDu xenograft tumor mouse model of human SCCHN, sHsp70 concentrations correlated with the tumor volume even in very small tumor sizes of ≈ 1 mm^3^[13]. Therefore, we speculate that sHsp70 levels might be useful in screening tests that aim to detect cancer at early stages or in small tumors or metastases also in humans. The FaDu xenograft model is frequently used to investigate the outcome of radiation therapy [18]. We could show that sHsp70 plasma levels were able to monitor local control of FaDu tumors in mice after irradiation with 30 Gy [13]. Therefore, a drop in serum sHsp70 levels might be able to predict the clinical outcome of radiotherapy in humans.

It has been shown that Hsp70 is actively released by viable, intact tumor cells and also at a lower level by dying cells [13]. Thus the detectable sHsp70 in the serum or plasma might be composed of two different sources. Other laboratories reported on increased levels of circulating sHsp70 up to several days after whole-body irradiation of mice bearing xenograft prostate tumors [19] that might be explained by dying cells. The slight increase in sHsp70 levels after radiation therapy might account for sHsp70 which is released by dying cells. Apart from dying cells viable tumor cells actively secrete large amounts of Hsp70 in vesicles. In order to evaluate the amount sHsp70, serum samples were derived of patients with SCCHN before start of any therapy. 18 out of 21 tumor patients showed significantly elevated sHsp70 levels in sera compared to healthy control subjects (Figure 1). We further could show that sHsp70 protein but not anti-Hsp70 antibody levels could be associated with the tumor volume in SCCHN patients (Figure 5A). Further analysis and larger studies with more patients will demonstrate whether diagnosis or clinical responses do correlate with sHsp70 levels [20].

The expression density of activatory NK cell markers which might be affected by the presence of sHsp70 protein in the serum was determined in peripheral blood lymphocytes of SCCHN patients before, during and after therapy. In recent studies we could show that especially the expression density of CD94 and NKG2D were found to be up-regulated upon stimulation with Hsp70 protein [21]. Herein, we could show that in all patients exhibiting sHsp70 the expression of NKG2D was significantly found to be up-regulated over time (Figure 6G). Future analysis will clarify whether the sHsp70 levels before therapy or changes of sHsp70 levels that are induced by therapy might be responsible for the increased expression of NKG2D on NK cells.

In summary, our data provide evidence that elevated sHsp70 protein levels in the blood of tumor patients are associated with the presence of primary malignant tumors in SCCHN patients. Furthermore, increased sHsp70 levels were found to be associated with increased tumor volumes. Since sHsp70 levels dropped in patients after removal of the tumor, we speculate that sHsp70 levels in the blood of patients might be useful not only for the detection of tumors but also for the monitoring of the therapeutic response to radiation therapy. Due to the finding that mHsp70 is associated with the capacity of tumor cells to actively release Hsp70 and due to the fact that mHsp70 positivity was determined on a broad variety of tumor entities such as colorectal, lung, pancreatic and prostate cancer [16,22-24], we assume that sHsp70 is also present in the serum of patients with other tumor entities. Future studies in larger cohorts and longer follow-up periods are necessary to determine the role of sHsp70 levels as a universal tumor biomarker.

## Competing interests

The authors declare that they have no competing interests.

## Authors’ contributions

MG performed experiments and drafted manuscript, HS collected blood samples and provided clinical data, CB performed ELISA experiments, MB collected tumor samples, BC collected tumor samples, MD collected blood samples, SB performed FACS analysis, KH performed ELISA, SL performed ELISA, VvP analyzed data, ES collected blood samples, TES analyzed data, MS performed collected blood samples, DS analyzed data, WS performed FACS analysis, SS performed FACS analysis, GM initiated the trial, analyzed data, drafted the Ms. All authors read and approved the final manuscript.
